# The Role of Transposable Elements in the Origin and Evolution of MicroRNAs in Human

**DOI:** 10.1371/journal.pone.0131365

**Published:** 2015-06-26

**Authors:** Sheng Qin, Ping Jin, Xue Zhou, Liming Chen, Fei Ma

**Affiliations:** 1 Laboratory for Comparative Genomics and Bioinformatics & Jiangsu Key Laboratory for Biodiversity and Biotechnology, College of Life Science, Nanjing Normal University, Nanjing, China; 2 The Key Laboratory of Developmental Genes and Human Disease, Ministry of Education, Institute of Life Science, Southeast University, Nanjing, China; 3 School of Chemistry and Biological Engineering, NanJing Normal University TaiZhou College, TaiZhou, China; University of Barcelona, SPAIN

## Abstract

MicroRNAs (miRNAs) are crucial regulators of gene expression at the post-transcriptional level in eukaryotes via targeting gene 3'-untranslated regions. Transposable elements (TEs) are considered as natural origins of some miRNAs. However, what miRNAs are and how these miRNAs originate and evolve from TEs remain unclear. We identified 409 TE-derived miRNAs (386 overlapped with TEs and 23 un-overlapped with TEs) which are derived from TEs in human. This indicates that the TEs play important roles in origin of miRNAs in human. In addition, we found that the proportions of miRNAs derived from TEs (MDTEs) in human are more than other vertebrates especially non-mammal vertebrates. Furthermore, we classified MDTEs into three types and found that TE head or tail sequences along with adjacent genomic sequences contribute to generation of human miRNAs. Our current study will improve the understanding of origin and evolution of human miRNAs.

## Introduction

Transposable elements (TEs) as important components of many genomes are able to mobilize and replicate in the host genomes [1]. There are two kinds of these elements: retrotransposons, and DNA transposons [2]. The retrotransposons can be further classified into three categories: long terminal repeat (LTR), long interspersed nuclear element (LINE) and short interspersed nuclear element (SINE). TEs were claimed to be an evolutionary force and were found to be related to epigenetic regulatory mechanisms [3–5]. In addition, TE sequences are able to provide TF binding sites during gene expression, and change regulatory networks of gene expression [6, 7].

The discovery of small RNA led to an emerging of prospect for uncovering the functions of TEs. Various small RNAs have been discovered, such as microRNAs (miRNAs), short interfering RNAs, piwi interacting RNAs and so on [8–10]. MiRNA is the first discovered small RNA [10]. MiRNAs are a class of short non-coding RNAs (approximately 22 nt) that are cleaved from longer (approximately 70 to 90 nt) precursor miRNAs (pre-miRNAs) [11, 12]. In animals, most miRNAs regulate gene expression by targeting mRNA-specific regions (known as miRNA-target sites) via partially complementary manner [13, 14]. These target sites are mainly located in 3’-untranslated regions (3’-UTRs) and complement with miRNAs via sequences of approximately 7 nt at the 5’ ends of miRNAs (known as ‘seed’ regions) [15, 16]. MiRNAs regulate gene expression by degrading mRNAs or repressing mRNA translation via recognizing their target sites [17]. Although many algorithms were developed to predict the target sites of miRNAs, the mechanism of miRNA recognition of target sites is not fully understood [15, 18–20]. Understanding the origin of miRNAs and their target sites will improve the rationale for developing algorithms to optimizing the prediction of miRNA-target genes.

TEs were claimed to provide a natural mechanism for the origin of new miRNAs and the targets of some miRNAs [21–25]. For instance, mir-28, mir-95 and mir-151 are derived from LINE-2 TEs, and mir-548 family is derived from Made1 TEs [21–23]. Alu elements of TEs could be targeted by almost 30 human miRNAs [25]. The hsa-mir-566 was found to be derived from Alu and 80% of its predicted target sites were claimed to be derived from TEs and related to Alu element [23]. However, it remains largely unknown what and how miRNAs originated from TEs in human.

In the current study, we provide evidences to show that TEs are important sources for the origin of miRNAs in human. Our results uncover the evolution of miRNAs derived from TEs in human and provide an insight into the mechanism of the origin of miRNAs.

## Materials and Methods

To identify the miRNAs derived from TEs (MDTEs), pre-miRNAs and their associated data were collected and analyzed by following steps:

Firstly, 6845 pre-miRNAs with chromosomal locations of eight vertebrates (*Danio rerio*, *Xenopus tropicalis*, *Gallus gallus*, *Bos taurus*, *Mus musculus*, *Macaca mulatta*, *Pan troglodytes* and *Homo sapiens*) were obtained from miRBase v20 [26]. The pre-miRNAs and their adjacent upstream and downstream 4,000 bp sequences were downloaded using the BioMart tool from Ensembl genome database (Release 68) [27].

Secondly, the pre-miRNAs and their adjacent sequences were used as the query sequences to identify TEs. TEs which locate on the query sequences were identified using the RepeatMasker program based on the repeatmasker libraries 20140131 [28, 29]. The Wu-blast program was used as the searching engine of RepeatMasker and the parameter—s was set to improve the accuracy of identification. The locations of TEs on query sequences were extracted from the “.out” file which is one of the output files of RepeatMasker.

Finally, the locations of TEs were compared with those of pre-miRNAs on query sequences. If a pre-miRNA overlapped with a TE on the query sequence, this miRNA was defined to be a MDTE. The proportions of overlap between pre-miRNAs and TE sequences were calculated for classification of MDTE types.

To identify a human MDTE which lose its sequence feature of TE and has homologous relations of miRNAs among human and seven other vertebrates were analyzed and human MDTEs without TE sequence feature were identified if a human miRNA showing non-overlapping with a TE but has homologies from several other vertebrates overlapping with same TE sequence.

To address whether different TE families have equal contributions to the origin of MDTEs, the proportions of MDTEs generated from different TE families were calculated and compared with the proportions of TE families in human genome. The proportions of MDTEs generated from different TE families were calculated following the procedure described above. The proportions of TE families in human genome were obtained from the published genome sequencing data [30]. Pearson's Chi-squared test was used to evaluate the significance of differences and *P*< 0.01 indicates that different TE families have different contributions to the origin of MDTEs significantly.

## Results and Discussion

### Identification of miRNAs derived from TEs in human and seven other vertebrates

**Fig 1:**
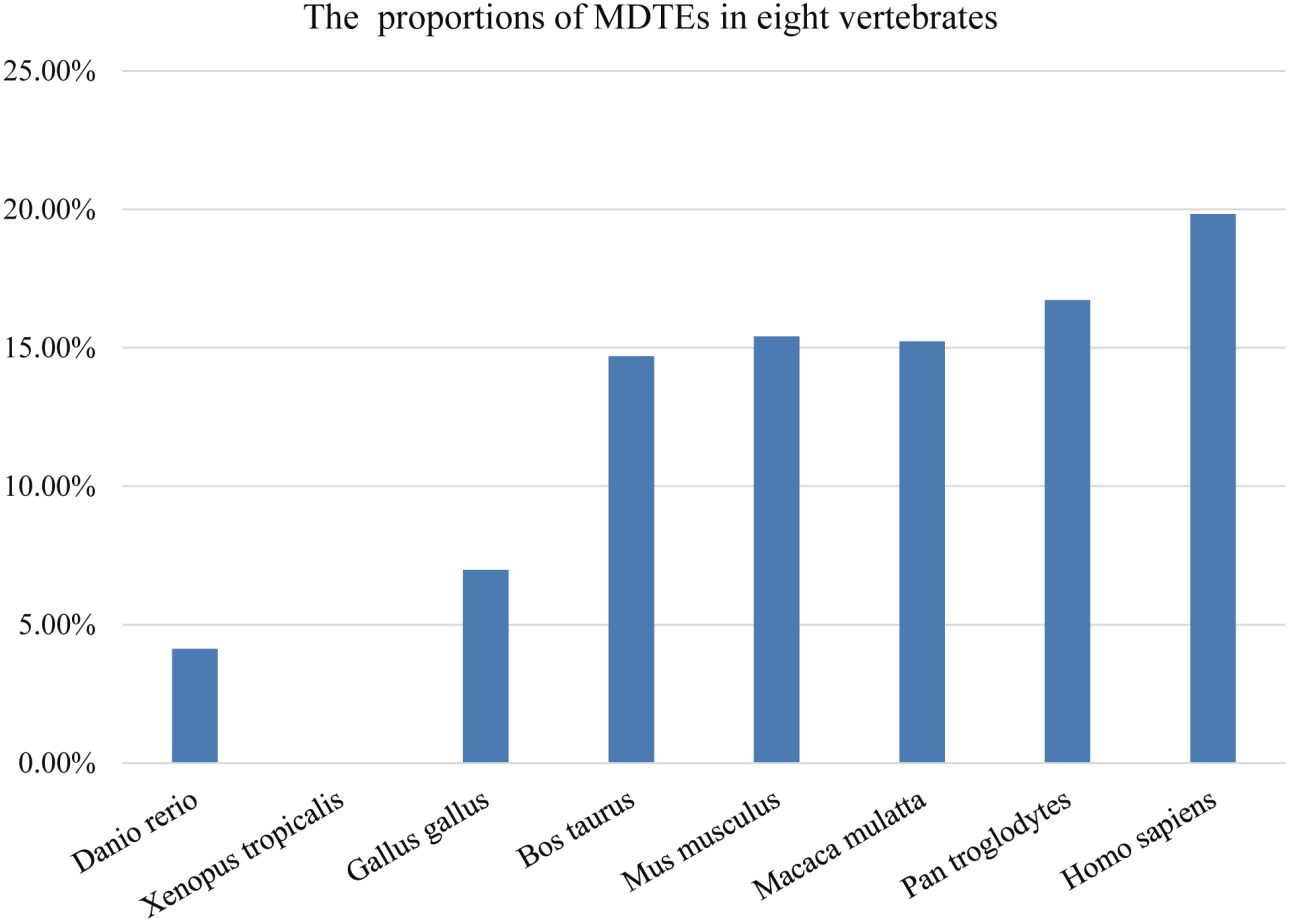
The proportions of MDTEs in human and seven other vertebrates.

The MDTEs were identified from human and seven other vertebrates (*Danio rerio*, *Xenopus tropicalis*, *Gallus gallus*, *Bos taurus*, *Mus musculus*, *Macaca mulatta* and *Pan troglodytes*). Surprisingly, non MDTEs were found in *Xenopus tropicalis*. Proportions of MDTEs in miRNAs increased with the evolution of vertebrates and the proportion in human was more than those in other analyzed vertebrates (Fig 1). Meanwhile, it was observed the proportions of MDTEs in miRNAs bear little relevance to the proportions of TEs in genomes. For example, although more than one-third of the genomes are made up by TEs in *Danio rerio* and *Xenopus tropicalis* [31, 32], the proportions of MDTEs in miRNAs are less than 5%. In comparison, TE sequences constitute 9% of genome in *Gallus gallus*, but 6.98% of miRNAs were MDTEs [33]. The MDTEs account for 19.84% of miRNAs in *Homo sapiens* and TE sequences make up 44.83% of its genome [30]. This observation might be due to the significant differences between the components of TEs in *Danio rerio* and *Xenopus tropicalis* and those in human and other mammals. This argument was supported, at least in part, by the observation that the major TEs are DNA types in *Danio rerio* and *Xenopus tropicalis* compared to retrotransposable elements in mammals [30–32, 34]. Given the contribution of TEs to miRNAs were negligible in *Drosophila* [35], MDTEs mainly present in genomes of human and other mammals. Information of MDTEs in human and seven other vertebrates was summarized and listed in Table 1.

**Table 1:** Summary of MDTEs in eight vertebrates.

	Unique MDTEs	Unique miRNAs	All miRNAs
*Danio rerio*	9	218	346
*Xenopus tropicalis*	0	154	189
*Gallus gallus*	48	688	734
*Bos taurus*	103	701	798
*Mus musculus*	169	1097	1186
*Macaca mulatta*	85	558	615
*Pan troglodytes*	96	574	656
*Homo sapiens*	338	1704	1872

Unique MDTEs are the numbers of miRNAs which are derived from TEs excluded multi-copy miRNAs. Unique miRNAs are numbers of all miRNAs excluded multi-copy miRNAs. All miRNAs are total numbers of miRNAs in a species.

When MDTEs were undergone homology analysis among *Danio rerio*, *Gallus gallus* and mammals, no homology of MDTEs among them were found. Fourteen MDTEs are conserved among five mammals and forty-seven MDTEs are conserved among primates. This finding implies that MDTEs are species-specific due to the difference of TEs among species.

### Analysis of MDTEs in human

To further investigate the pattern of MDTEs in human, 1872 miRNA gene sequences of *Homo sapiens* collected from the miRBase v20 were mapped to the human genome and analyzed. In total, 386 MDTEs which completely or partly overlap with TEs show unique relationships to their related TEs. It can be demonstrated via observing the origin of multi-copy MDTEs or MDTE families. Each copy in multi-copy MDTEs or each member of a MDTE family was found to be originated from the same TE. For example, six copies of hsa-mir-3118 in human genome are all partly derived from LINE/L1PA13 and a large miRNA group, hsa-mir-548, is derived from DNA/MADE1 element. Taken together, our findings suggested that a MDTE and its homologies are derived from the same TE. S1 Table lists detailed information of all MDTEs.

**Fig 2:**
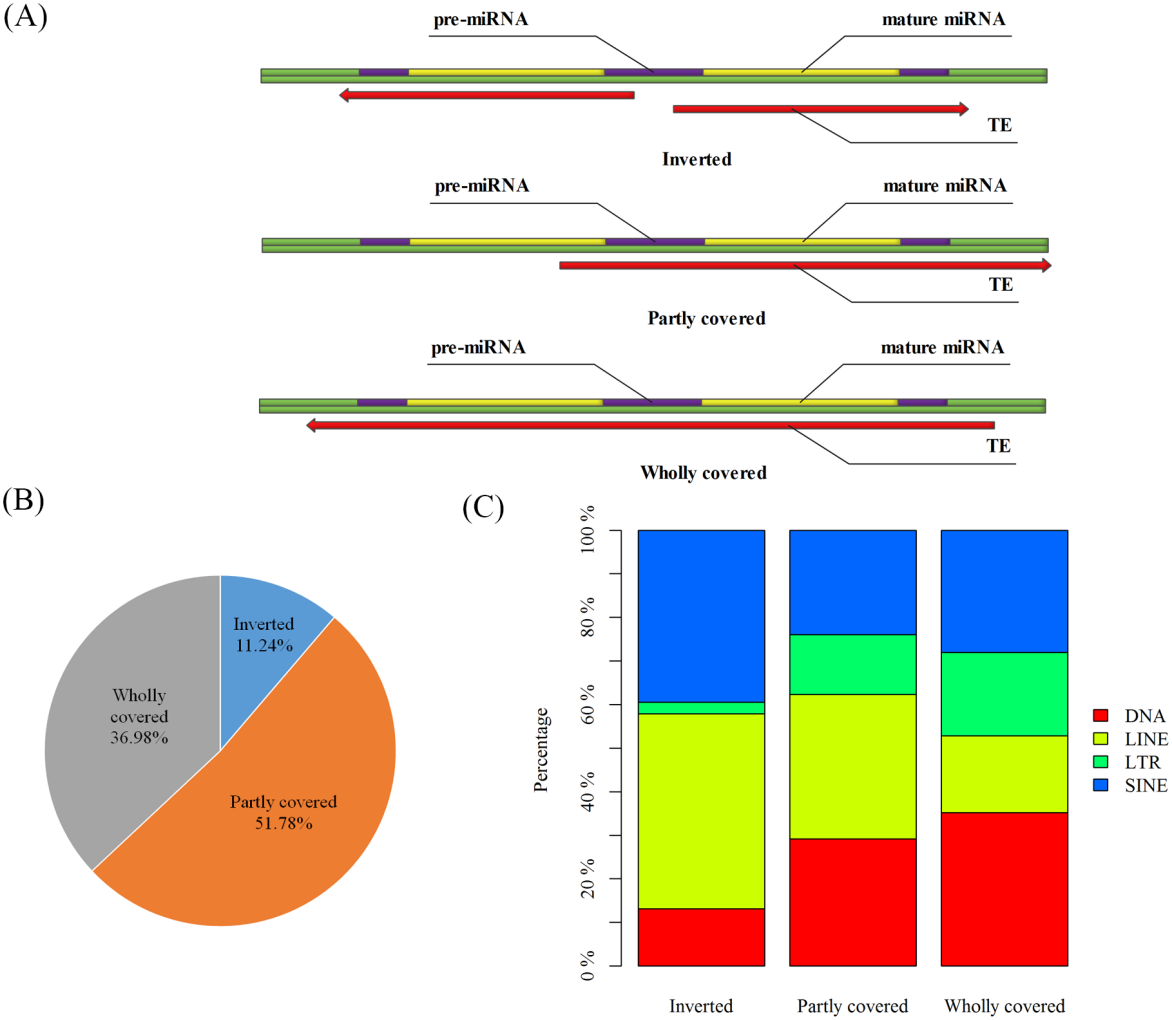
Analysis of three types of MDTEs. **(A) Three types of MDTEs. Type I Inverted**—The pre-miRNA is derived from two inverted TEs. **Type II Partly overlapped**—One arm is a TE while the other is the complementary sequence of that TE. Part of the pre-miRNA is derived from a TE, but another part is without TE features. **Type III Wholly overlapped**—The pre-miRNA wholly overlaps with a TE. Genome sequence is marked with green bar, pre-miRNA with purple bar and mature miRNA with yellow bar. Red arrow indicates the direction of TE sequence. **(B) Percentage of three types of MDTEs. (C) The proportion of TE families in three types of MDTEs**.

When multi-copy MDTEs were excluded, 338 unique MDTEs (UMDTEs) were identified and can be classified into three types (Fig 2A): Type I UMDTEs derived from inverted TE sequences, Type II UMDTEs with sequences partly overlap with TE sequences that are not inverted, and Type III UMDTEs with sequences wholly overlap with TE sequences.

MiRNAs have been identified in various organisms with rapidly increasing number in databases. In humans, except for multi-copies, approximately 19.84% (338/1704) of miRNAs overlapping with TEs are regarded as UMDTEs. Inverted TEs have been claimed as an important configuration of miRNA origin [21, 22]. Consistently, 11.24% of UMDTEs were found to be derived from inverted TEs, 36.98% of the UMDTEs were found to be derived from whole TEs (Fig 2B). It might be due to the abundance of similar fragments and palindromic structure in TEs [4, 36]. These fragments provide the potential to form the hairpin structure of miRNAs. Interestingly, approximately 51.78% of UMDTEs partly overlap with TEs (Fig 2B).

Four TE classes (SINE, LINE, LTR and DNA) have different contributions for three types of MDTEs (Fig 2C). In human, SINEs, LINEs, LTR retroposons and DNA transposon copies comprise 13%, 20%, 8% and 3% of the genome sequences (The proportions of four TEs as a whole: SINE:29.55%; LINE:45.45%; LTR:18.18%; DNA:6.82%;) [30]. SINE and LINE (39.47% & 44.74%) are the major contributors for Type I UMDTEs. The bulk of Type II UMDTEs is composed of LINE, DNA and SINE (33.14%, 29.14% and 24.01%). In Type III UMDTEs, DNA and SINE are main resources (DNA:35.20%, SINE:28.00%), while LTR and LINE take up 19.2% and 17.6% respectively.

**Fig 3:**
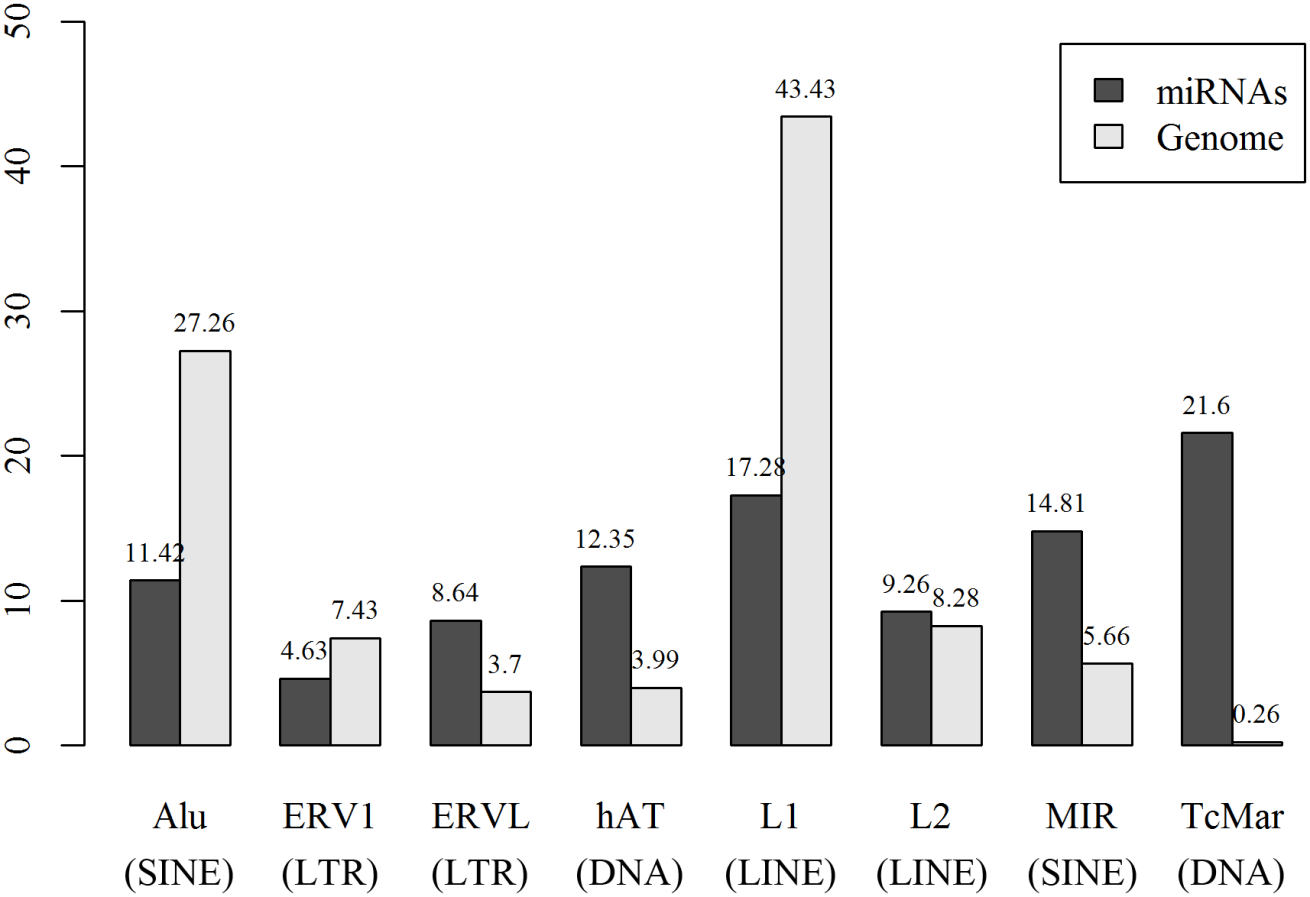
Percentage of MDTEs and different TE families in human genome. The sum of relative percentage is 100% for each group.

Compared to frequencies of TE families in human genome, TE families show different contributions to human MDTEs (Pearson's Chi-squared test: χ^2^ = 49.65, *P* = 1.69e^-8^, Fig 3). Consistent with the result of previous work [23], MIR (SINE) and DNA elements including TcMar and hAT families contribute more to origin of miRNAs compared to their frequencies in human genome. In comparison, Alu (SINE), L1 (LINE) and ERV1 (LTR) families less overlap with miRNAs. Unexpectedly, ERVL (LTR) family shows more overlap than expected regarding its frequency in human genome.

### Type II MDTEs are derived from TEs via two patterns in human

**Fig 4:**
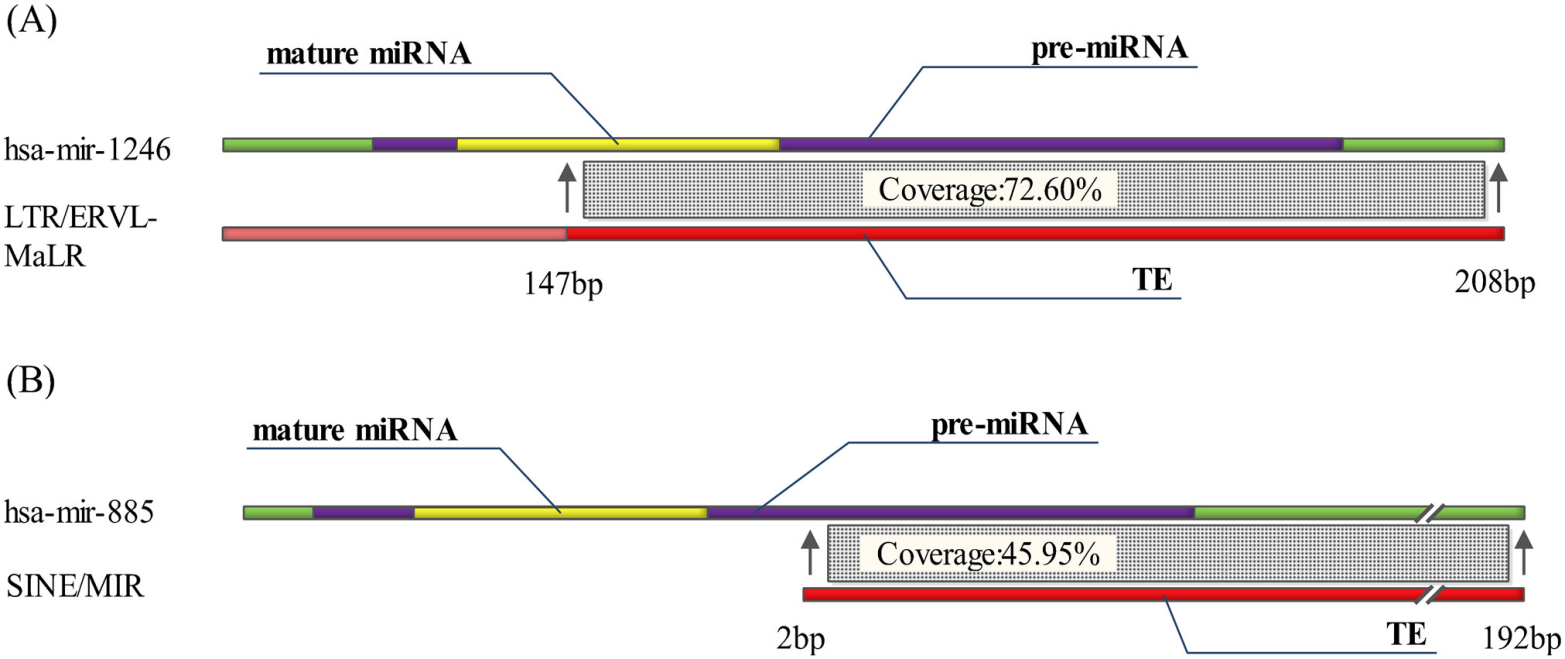
Two patterns of Type II MDTEs. **(A) Pattern I MDTEs**. The MDTEs lost their TE features of the wholly overlapped type. For example, 72.60% of hsa-mir-1246 with its downstream sequence was derived from 147 bp to 208 bp of MLT1M. The sequence before 147 bp has lost its TE features. **(B) Pattern II MDTEs**. TEs take up half of the miRNAs from their head or tail. For example, the 3’ arm of hsa-mir-885 with its downstream sequence is derived from 2 bp to 192 bp of AluSc. The 5’ arm is derived from non-TE sequence.

In UMDTEs, 51.78% miRNA belongs to Type II MDTEs which partly overlap with TEs. Type II MDTEs was found to be generated by two patterns: Pattern I in which MDTEs loss their TE sequence features from whole TE sequences, and Pattern II in which MDTEs with a part of the pre-miRNA are derived from the head or tail of TEs (Fig 4). In Pattern I, it is evident to observe MDTEs in miRNA homologies or multi-copy miRNAs obviously. The overlap between TEs and MDTEs in pattern I is reduced from 100% to 30% or even less (Table 2).

**Table 2:** Extent of overlap in the same miRNA homologies or multi-copy miRNAs.

miRNAs	TEs	Overlap	miRNAs	TEs	Overlap
hsa-mir-1289-1	MER5A	100.00%	hsa-mir-548ab	MADE1	100.00%
hsa-mir-1289-2	MER5A	78.38%	hsa-mir-548u	MADE1	97.53%
hsa-mir-3118-1	L1PA13	53.33%	hsa-mir-548q	MADE1	96.00%
hsa-mir-3118-2	L1PA13	53.42%	hsa-mir-548f-3	MADE1	95.40%
hsa-mir-3118-3	L1PA13	53.33%	hsa-mir-548e	MADE1	90.91%
hsa-mir-3118-4	L1PA13	41.33%	hsa-mir-548m	MADE1	90.70%
hsa-mir-3118-5	L1PA13	40.79%	hsa-mir-548l	MADE1	89.53%
hsa-mir-3118-6	L1PA13	41.33%	hsa-mir-548v	MADE1	88.75%
hsa-mir-378a	MIRc	65.15%	hsa-mir-548g	MADE1	87.64%
hsa-mir-378b	MIRc	61.40%	hsa-mir-548az	MADE1	84.21%
hsa-mir-378d-1	MIRb	42.59%	hsa-mir-548ap	MADE1	81.25%
hsa-mir-378d-2	MIRc	44.90%	hsa-mir-548x-2	MADE1	80.00%
hsa-mir-378e	MIRc	31.65%	hsa-mir-548a-1	MADE1	77.32%
hsa-mir-378f	MIRc	50.00%	hsa-mir-548f-4	MADE1	71.43%
hsa-mir-378g	MIR3	100.00%	hsa-mir-548k	MADE1	68.10%
hsa-mir-378h	MIRc	100.00%	hsa-mir-548i-1	MADE1	53.02%

Compared with Pattern I MDTEs, which account for 77.14% of Type IIMDTEs, TEs form one arm of the pre-miRNAs in Pattern II MDTEs. In this condition, the TEs were inserted in the proximity of appropriate sequences that are similar to the complementary sequences of the TE head or tail to form the hairpin structure of pre-miRNAs, such as hsa-mir-326, hsa-mir-421 and hsa-mir-619 (S2 Table). For Pattern II MDTEs, the mature miRNAs are derived from not only the internal portion of TEs but also non-TE sequences that were complementary with the head or tail of TEs.

Two origin mechanisms of MDTEs can be found across three types of MDTEs. In Type II and Type III MDTEs, some miRNAs were generated via being taken into genomes by TEs and passed on to new species after species differentiation. In Type I and Type II MDTEs, some miRNAs are generated by TEs from current genome.

### Identification and characterization of human MDTEs without TE sequence features

About 19.84% miRNAs wholly or partly overlap with TE sequences in human, but the origin of other miRNAs is not very clear. To identify those MDTEs losing their sequence features of TEs, the miRNAs which do not overlap with TEs in human were analyzed and compared with their homologies in other vertebrates. Twenty-three miRNAs were identified as human MDTEs which do not overlap with TEs, while their homologies either wholly or partly overlap with same TE sequence in other species (S3 Table). Although these MDTEs just account for 1.35% of all miRNAs in human, it implies that more miRNAs than expected may be derived from TE sequences in vertebrates.

## Conclusion

In summary, we found that TE is an important origin source of human miRNAs. MiRNAs can be brought into genomes during the insertion of TEs or generated by TE sequences via particular mechanisms in current genome. When MDTEs fixed in the genome, sequence features of TE of MDTEs might be lost during the evolution. The observation that some MDTEs partly overlap with TE sequences and some MDTEs do not overlap with TEs implies that there are more MDTEs in genomes of vertebrates than what we previously believed. Our findings provide an insight into the origin and evolution of miRNAs.

## Supporting Information

S1 Table MDTEs overlap with TEs. (XLSX)

S2 Table Two patterns of Type II MDTEs. (XLSX)

S3 Table MDTEs do not overlap with TEs but their homologies overlap with TEs. (XLSX)
